# Quantitative phase imaging and Raman micro-spectroscopy applied to Malaria

**DOI:** 10.1186/1746-1596-8-S1-S42

**Published:** 2013-09-30

**Authors:** Jacques Klossa, Benoit Wattelier, Teddy Happillon, Dominique Toubas, Lucie de Laulanie, Valérie Untereiner, Pierre Bon, Michel Manfait

**Affiliations:** 1 TRIBVN, 39, rue Louveau, 92320 Châtillon, France; 2 Phasics, Campus de l'Ecole Polytechnique, 91128 Palaiseau, France; 3 MEDyC FRE/CNRS 3481, 51096 Reims, France; 4 CHU de Reims, Laboratoire de parasitologie-mycologie, 51100 Reims, France

## Background

Malaria is due to parasitism of red blood cells (RBC) by protozoan parasites of the genus Plasmodium. Three main parameters have to be determined for patient treatment: parasite species, the rate of infected blood cells (parasitemia), and development stage. Even if a series of laboratory techniques are available, a suited treatment needs microscopy skills [1]. Microscopic observation needs a specialist and is time consuming (e.g. observation of hundreds fields of view at 100x immersion objective) and automating 100x slide scanning of white light imaging of thin film stained blood smears is not straightforward.

Seeking for an easy to automatize alternative, we thought that combining two microscopy techniques: quantitative phase imaging for quick detection and Raman micro-spectroscopy for molecular characterization could appear to be an efficient multipurpose solution.

Raman micro-spectroscopy is a good candidate to identify molecular species in microscopic. Laser light is focused in a tiny volume. Due to molecular vibrations, part of the light is non-linearly scattered to longer (Stokes) or shorter (Anti-Stokes) wavelength. The wavelength shift is directly linked to the vibration energy. The scattered light spectrum contains lines typical of the molecular binding in the focused volume. However imaging is very time consuming as each pixel needs to be acquired individually (between 1 and 10s per pixel).

Quantitative phase imaging is an imaging technique that measures the optical path difference of light travelling through different part of a semi-transparent medium. If a biological tissue has local different index of refraction, we obtain an image that reflects these index changes. This kind of maps usually has a contrast more than two orders of magnitude higher than bright field microscopic images. In addition, they give information about the relative index change in the tissue.

Combining a morphological technique suited for rare events detection with a molecular technique suited for local molecular signature acquisition could provide an easy way to address automation of parasite parameters acquisition.

In the following sections, we present our first level attempt for proof of concept.

## Material and methods

### Sample preparation and hardware

We used for our experiments the solution developed during the IHMO program (see "Label free technologies: Raman micro-spectroscopy and multi-spectral imaging for Lymphocyte classification" in the current publication). It uses the Calopix TRIBVN^TM^ software platform integrated to the hospital workflow and off the shelves components NIKON FN1^TM^ microscope fitted with a Raman micro-spectroscopy (RMS) HORIBA module whose excitation source is a 532nm diode laser. In addition a commercial quadriwave lateral shearing interferometer (QWLSI Phasics SID4Bio^TM^) is plugged on one of the microscope exit port; it measures the quantitative phase shift in the visible spectral band. This is an easy-to-integrate and compact solution (dimension of a simple camera) and gives a 300x400px^2^ (px=pixel) phase and intensity image with a lateral pitch of p=29.6 µm in the image plane, which corresponds to 0.74µm/px for 40x magnification and 0.197µm/px for 150x magnification.

Blood smears are prepared on a standard glass slide with no slide cover and without any previous staining to avoid contamination of the recorded Raman spectra with fluorescence contribution. Spectra are acquired on infected RBC and finally, slides are stained with May-Grünwald Giemsa for morphological validation.

### Quantitative phase imaging

**Figure 1:**
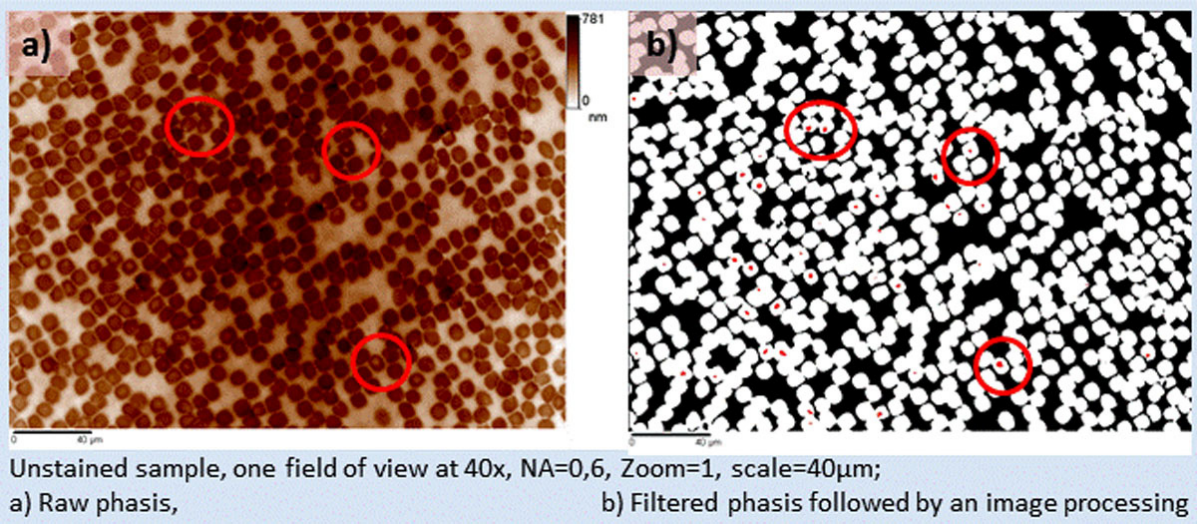
parasite characterization: a) microscope image, b)quantitative phase image, c) RMS spectra comparison between infected RBC and healthy RBC after hemoglobin mean spectrum subtraction

**Figure 2:**
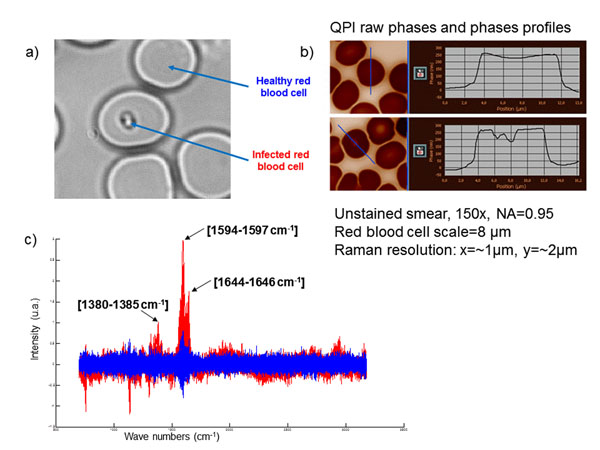
blood smear exploration and parasite detection with quantitative phase imaging

The presence of the parasite inside the RBC implies a synthesis of hemozoin (complex molecular structure) from hemoglobin. These two molecules present a different refractive index; therefore the parasite should appear with a different quantitative phase inside the RBC. We have checked this assumption at high magnification (150x) and we found out that the parasite induces lower phase values (figure 1). Afterward, we studied large fields of view at lower magnification: 40x proved to be efficient: see a typical field of view figure 2. In order to automatically detect the infected cells, we first applied a high pass filter. This removes the slowly varying cell shape while keeping the plasmodium image. Then low phase values due to the plasmodium were detected by three-level segmentation. One level corresponds to the extracellular medium; the second level corresponds to the RBC cytoplasm containing hemoglobin and the third one to the hemozoin. The second level segmentation leads to the number of the RBC contained in the field. The third level segmentation gives the number of plasmodia and thus of infested RBCs. The ratio between these two numbers is the parasitemia. We have studied 10 fields of a single blood smear containing falciparum parasites.

### Raman micro-spectroscopy

Raman spectra are recorded on parasite from infected RBC localized thank to QPI at 40x. Raw spectra need first a pre-treatment phase to make them eligible in the classification process. Indeed, the presence of hemoglobin into the cells, and more precisely the fact that hemoglobin contains cyclic structures, implies distortions of the baseline of each spectrum. This distortion effect is called a fluorescence background. To correct the spectra, a function based on a polynomial estimation and correction of the baseline is used for each spectrum independently of each other. Then, the normalization function, standard normal variate, is used on each spectrum. This function eliminates the variation of the absolute values into the spectra, and makes them comparable avoiding scale differences. Then, a first classification could be realized. For such purpose, we used the classical Hierarchical Clustering Analysis (HCA). HCA is based on the Euclidean distance between each observation (each spectrum) and from these distances a hierarchical binary tree is created, putting forward the different groups of spectra. Representative peaks of the hemozoin biocrystal, previously identified in literature, are taken into account to classify the spectra, by reducing them to the corresponding region of interest. In a second classification, and to improve the first results, a representative spectrum of pure hemoglobin, which has been obtained by averaging RBC spectra from other samples, has then been estimated and subtracted from each recorded spectrum, thanks to a mean squared based function.

## Results and discussion

### Quantitative phase imaging results

QPI analytical process detected an average of 640 blood cells per field at 40x (standard deviation of 23) and an average of 4.1% of infected cells (standard deviation is 1.1%) for sample labeled as a 3.5% parasitemia.

### Raman micro-spectroscopy results

The first classification realized considered the pre-treated spectra reduced to the representative peaks of the hemozoin biocrystal (i.e. 1380-1385, 1594-1597 and 1644-1646 cm-1). After a HCA classification the full 35 spectra obtained from sane red blood cells were classified in the first cluster of the HCA dendrogram, but only 20 spectra on the 34 registered on parasites were present in the second cluster, which gave a sensitivity of 100% and a specificity of 58.8%.

To improve these first results, a second classification was realized, considering every spectrum without the contribution of the hemoglobin signal as mentioned previously. Then, the full 35 spectra from the parasites where present in the first class of the dendrogram and the full 34 spectra from sane red blood cells were classified into the second cluster, giving a sensitivity and a specificity of 100%.

## Discussion

This study proved the ability of QPI to detect RBC and infected RBC at low magnification without any previous staining. Each detected infected RBC can then be easily confirmed with RMS spectra acquisition. Making such global process easy to standardize and easy to automate.

The proposed concept is very efficient on this practical use case thanks to QPI efficiency that allows quick retrieving of a representative cell population without any previous staining. However during further studies, detection potential of poorly differentiated parasite should be compared to 100x classical immersion microscopy on stained sample.

RMS highlights thin molecular information and has been 100% sensible and specific in the current study. However, the presence of hemozoin into the parasite could be variable in quantity or even nonexistent which in further studies could lead to spectra misclassifications. Finally, it will also be necessary to differentiate species: this should be assessed on following studies while using different high resolution (150x) techniques on infected cells: i) RMS spectra, ii) optical volume measure with QPI and iii) multi-spectral classification (see IHMO project).

Following further investigations, combination of quick detection with Quantitative Phase Imaging and selective molecular imaging with Raman Micro-Spectroscopy has a good potential to automate parasite detection and counting. Such toolkit could be applied to any kind of parasite, bacteria or bacteria inside vesicles and it also could be extended to other cytology use cases and likely to the field of histopathology.

## List of abbreviations

HCA: ; QPI: Quantitative phase imaging; Px: pixel; QPI: Quantitative phase imaging; QWLSI: ; RBC: Red blood cell; RMS: Raman micro-spectroscopy

## Competing interests

The authors declare that they have no competing interests.

## Authors’ contributions

• TH carried out the acquisitions, pretreatments, the highlighting of relevant information into the spectra, the classification of the data and drafted the extended abstract.

• VU realized the acquisitions of the Raman spectra and drafted the extended abstract.

• PB, LdL and BW developed the multi-spectral classification method and produced the multi-spectral classification.

• JK, BW and MM both designed and managed the study.

• DT selected the infected patients, provided the corresponding samples and patient’s data, and did microscopy assessments.
